# Comprehensive evaluation of RNA-seq quantification methods for linearity

**DOI:** 10.1186/s12859-017-1526-y

**Published:** 2017-03-22

**Authors:** Haijing Jin, Ying-Wooi Wan, Zhandong Liu

**Affiliations:** 1 Graduate Program in Structural and Computational Biology and Molecular Biophysics, Baylor College of Medicine, One Baylor Plaza, Houston, 77030 TX USA; 2 Department of Molecular and Human Genetics, Baylor College of Medicine, One Baylor Plaza, Houston, 77030 TX USA; 3 Department of Pediatrics-Neurology, Jan and Dan Duncan Neurological Research Institute, Baylor College of Medicine, 1250 Moursund St., Suite 1325, Houston, 77030 TX USA

**Keywords:** RNA-seq, Deconvolution, Linearity

## Abstract

**Background:**

Deconvolution is a mathematical process of resolving an observed function into its constituent elements. In the field of biomedical research, deconvolution analysis is applied to obtain single cell-type or tissue specific signatures from a mixed signal and most of them follow the linearity assumption. Although recent development of next generation sequencing technology suggests RNA-seq as a fast and accurate method for obtaining transcriptomic profiles, few studies have been conducted to investigate best RNA-seq quantification methods that yield the optimum linear space for deconvolution analysis.

**Results:**

Using a benchmark RNA-seq dataset, we investigated the linearity of abundance estimated from seven most popular RNA-seq quantification methods both at the gene and isoform levels. Linearity is evaluated through parameter estimation, concordance analysis and residual analysis based on a multiple linear regression model. Results show that count data gives poor parameter estimations, large intercepts and high inter-sample variability; while TPM value from Kallisto and Salmon shows high linearity in all analyses.

**Conclusions:**

Salmon and Kallisto TPM data gives the best fit to the linear model studied. This suggests that TPM values estimated from Salmon and Kallisto are the ideal RNA-seq measurements for deconvolution studies.

**Electronic supplementary material:**

The online version of this article (doi:10.1186/s12859-017-1526-y) contains supplementary material, which is available to authorized users.

## Background

Next-generation sequencing based technology for RNA profiling (RNA-seq) has become the predominant method to quantify the transcript abundance in cells. Compared to microarray technology, RNA-seq offers broader quantification range and enables the detection of novel transcripts [1]. However, due to the fragmentation of sequencing material, there is greater complexity in quantification and analysis of RNA-seq data [2]. Current state-of-the-art quantification tools for RNA-seq data can be divided into two major categories [3]: alignment-based and alignment-free. Alignment-based quantification methods will first map each sequenced reads to a reference genome or transcriptome and then estimate the abundance of transcripts based on the alignment. Alignment-free quantification methods rely on light-weight pseudo-alignment in k-mer space to quantify the transcript abundance. An analytic challenge raised from these quantification methods is that different method generates abundance measurements in different units, including counts, FPKM (Fragments Per Kilobase of transcript per Million mapped reads), RPKM (Reads Per Kilobase of transcript per Million mapped reads), and TPM (Transcripts Per Million) [4]. Furthermore, various transformation strategies can be applied to quantification values in purpose of specific downstream analysis like differential gene expression analyses [5] or novel splicing site detection [6]. Although several studies have provided assessment of analysis tools for RNA-seq data, little consensus on the optimal analysis pipeline is obtained [4, 6–9]. Deconvolution is a mathematical process used to extract constituent elements from a mixture of multiple signals [10]. In the field of biomedical research, deconvolution is widely applied to retrieve cell-type or tissue specific gene expression profiles from heterogeneous tissue samples. Most deconvolution algorithms in the literature assume a linear model [10–17], in which the expression signal of the mixture is a weighted sum of the expression for its constitutive cell types. Previous analysis has shown the necessity of using anti-log expression microarray data to avoid unwanted bias introduced by non-linear transformation [18]. However, no study has assessed the linearity of transcript abundance in RNA-seq data. Therefore, in this study, we conducted a comprehensive comparison of seven RNA-seq quantification methods on the linearity of the estimated abundance using a deep sequencing dataset where RNA samples were mixed at known proportions. Our results will provide a good recommendation to researchers considering deconvolution on RNA-seq data.

## Results

### Data

We employed the benchmark dataset used to assess RNA-seq measurement performance in different application sites and platforms from the Sequencing Quality Control (SEQC) project [19]. In order to have minimal inter-sample variability in the linearity evaluation analyses, we included samples from the same platform (Illumina HiSeq 2000) and same sequencing center (NVS). Specifically, raw sequenced reads for four biological replicates of four types of samples (A, B, C, D) were obtained; where sample A is derived from universal human reference RNA, sample B is derived from human brain reference RNA, sample C is obtained by mixing A and B in ratio 3:1, and sample D is obtained by mixing A and B in ratio 1:3. Out of 12 samples from A, B and C, nine samples have about eighty million pairs of raw reads and three samples have double the depth. Overall, the mappability of all the samples is around 70–80%. A brief summary about the samples is given in Additional file 6: Table S1.

### Quantification methods

We performed a literature survey and selected seven prevalent quantification methods for comparison. To increase the comparability of the estimated transcript abundance, all the alignment-based quantification methods were applied on mappings processed with Tophat2 [20]. HTSeq-count [21] provides the number of reads/fragments mapped unambiguously to a single feature, referred as count. Cufflinks [22], which is also the most popular quantification method, uses comparative algorithm assembly to produce minimal set of transcript supported by the transcript alignment. The resulting transcript abundance is measured in FPKM. EdgeR [5] models count data based on an overdisposed Poisson model and uses an empirical Bayes procedure to moderate the degree of overdispersion across genes, which is intended for downstream gene expression analysis. RSEM [3] uses a generative model of RNA-Seq reads and the expectation-maximization (EM) algorithm to estimate abundances of transcript feature. The two alignment-free based quantification methods, Kallisto [23] and Salmon [24] apply pseudo-alignment to find potential transcript origins of RNA-seq reads.

### Data distribution

**Fig. 1:**
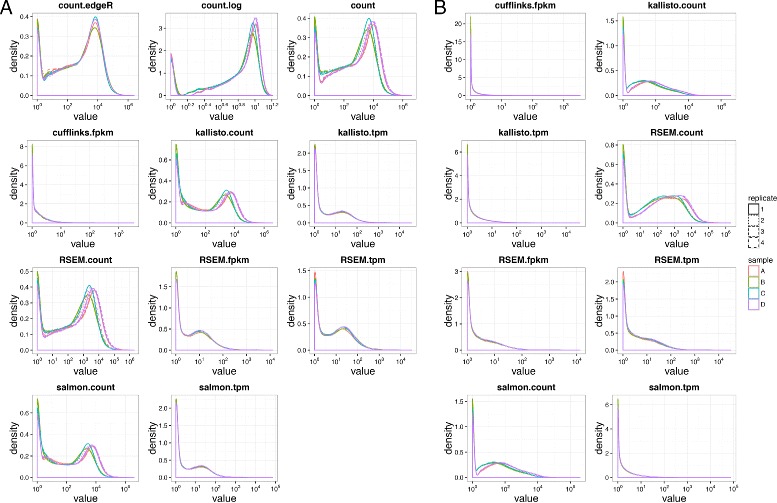
Distribution of quantifications at gene level (**a**) and isoform level (**b**)

To our surprise, the distribution of the estimated abundance from each quantification method results in distinct distribution at both the gene level (Fig. 1 a) and isoform level (Fig. 1 b). Except Cufflinks FPKM, all distributions contain two sharp peaks for abundance at gene level (Fig. 1 a). While count values are normalized to TPM or FPKM, the second peak is weakened and results in a smoother curve. Although the scaling/normalization factors based on library size and gene length used in TPM and FPKM will explain the reduced range of the quantifications, it cannot explain the reduced height and smoothened second peak in the distribution. For example, logarithm transformation reduces the range of HT-Count quantifications in scale, without weakening the second peak (Fig. 1 a). The sharpness of the second peak observed at the gene level diminished at isoform level for all quantification methods (Fig. 1 b). Nevertheless, the distribution pattern at both gene and isoform levels remains consistent for the same quantification method.

### Assessment of linearity

**Fig. 2:**
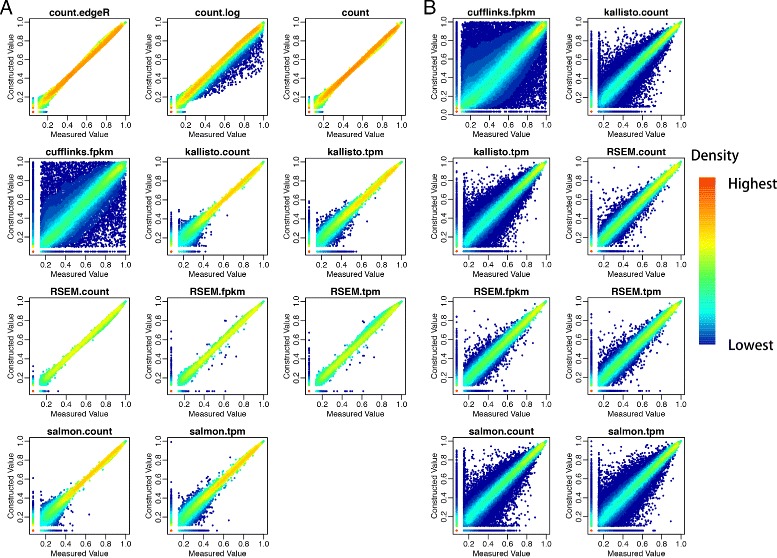
Concordant analysis between rank of quantifications of $0\text {.}75\times \bar {A}+0\text {.}25\times \bar {B}$(*Constructed Value*) and $\bar {C}$ (*Measured Value*) at gene level (**a**) and isoform level (**b**). Rankes were normalized by the number of quantifications in each plot

**Fig. 3:**
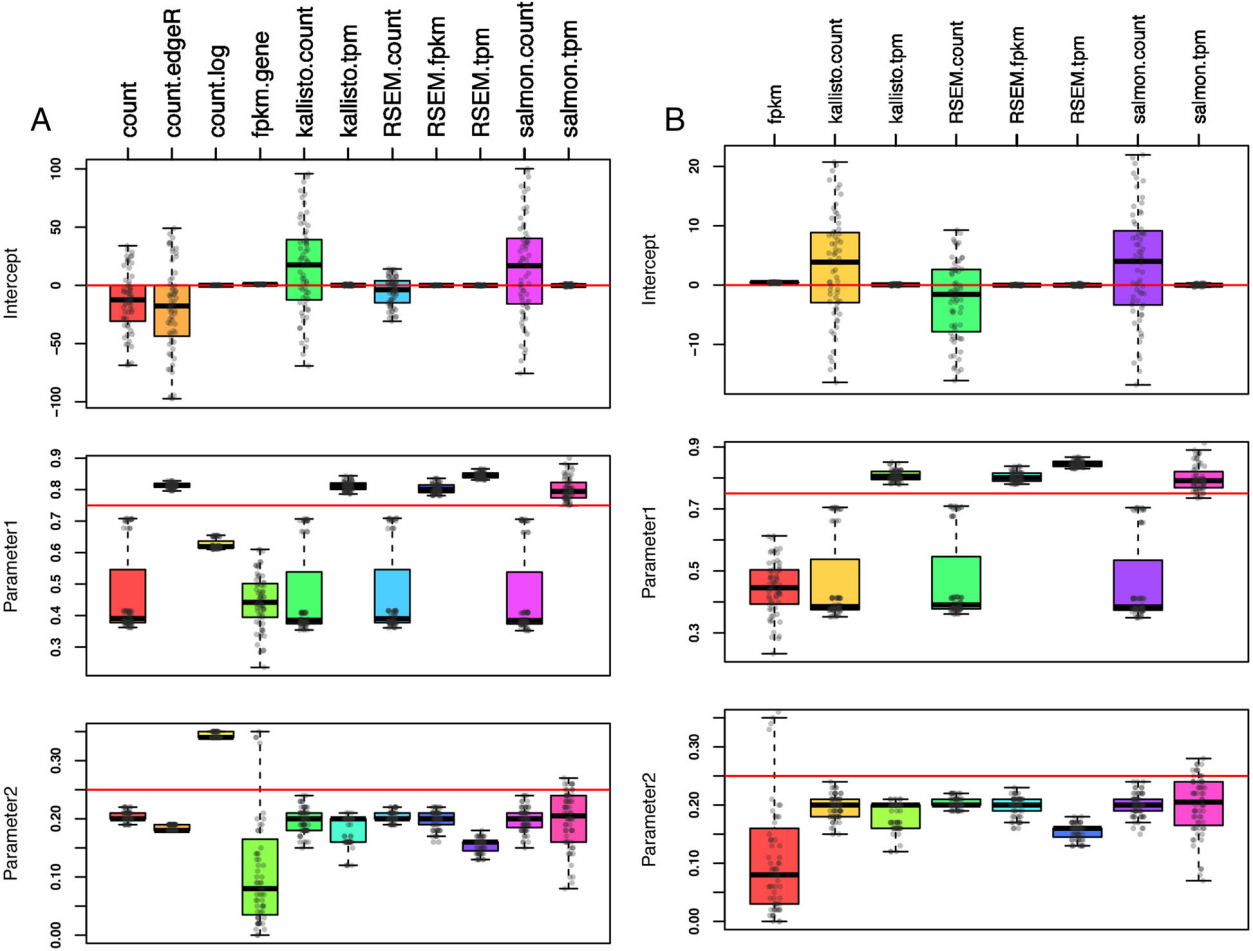
Jitter boxplot of estimated coefficients and intercepts from linear model *C*∼*m*×*A*+*n*×*B*+*ε* at gene level (**a**) and isoform level (**b**). *Red line* indicates expected estimates if *C*, *A* and *B* satisfy linear assumptiotn

To evaluate the linearity of the RNA-seq data produced by different quantification methods, we used a multiple linear regression model: *C*∼*m*×*A*+*n*×*B*+*ε*. Since sample *C* is derived by mixing sample *A* and *B* at ratio of 3:1, the expected values for the parameters *m*, *n*, and *ε* should be 0.75, 0.25, and zero respectively. We reason that if the RNA-seq data are linear, the fitted multiple linear regression model will provide precise estimation for the parameters *m*, *n*, and *ε*. Since there are four biological replicates for each sample types (*A*, *B*, *C* or *A*, *B*, *D*), there is a total of 64 possible models to be fitted. We fitted all 64 models and studied the performance based on the average of the parameters and predicted values. As shown in Fig. 2, the true value of *C* is linearly correlated to the fitted values from models with known mixture proportion 0.75 and 0.25 as parameters in all quantification methods. The linear relationships are especially pronounced at the gene level (Fig. 2 a). Although there are more data points away from the diagonal at the isoform level (Fig. 2 b), the high density along the diagonal represents strong linear relationships. Results on the estimated coefficients and intercepts from the 64 models show that count data produce large intercept values at both the gene level and isoform level (Fig. 3, top panel). The intercept values are also sensitive to the counts data from different samples fitted, resulted in highly variable results across models of different samples. FPKM values from Cufflinks give the largest variation in estimated m and n among all non-count quantifications. Overall, TPM and FPKM quantifications reported by Salmon, Kallisto and RSEM resulted in the best estimate of coefficients with small 95% confidence intervals. Similar analyses on isoform level abundance give the same results as observed in gene level data (Fig. 3 b, middle and bottom panel).

We observed that the estimation of m resulted in two clusters from count data (Fig. 3, middle panel): one close to true value of 0.75 and another center around 0.4. We found that this phenomenon is due to the library size difference between samples. Specifically, results from cluster close to 0.75 are from 16 combinations with the second replicate of sample *A*. From Additional file 6: Table S1, we can find that the second replicate of *A* is the only sample among the four replicates to have similar library size with other samples of *C* and *B*. From this result, we could conclude that normalization of quantified abundance is essential to eliminate the inter-sample variability to meet the linear assumption of deconvolution analysis. To further assess the linearity of the RNA-seq quantifications, we evaluated the fitted model’s prediction through three analyses: 1) a concordance analysis between measured *C* and fitted value $\hat {C}$, 2) a receiver operating characteristic (ROC) curve-like analysis on the absolute residual values, and 3) residual analysis for rescaled model. Similar to parameter estimation analysis mentioned above, linear regression is performed on 64 models, which is constructed upon combinations of 4 replicates in each sample, then estimated value $\hat {C}$ and residuals are averaged for final analysis.

**Fig. 4:**
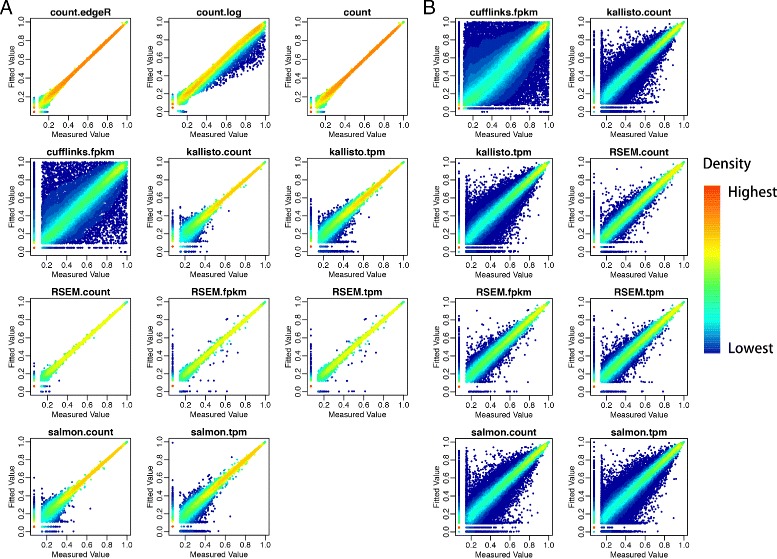
Concordant analysis between rank of estimated quantifications and rank of measured abundance value at gene level (**a**) and isoform level (**b**). The *fitted value in the y-axis* is estimated from model *C*∼*m*×*A*+*n*×*B*+*ε*. Ranks were normalized by the number of quantifications in each plot

**Fig. 5:**
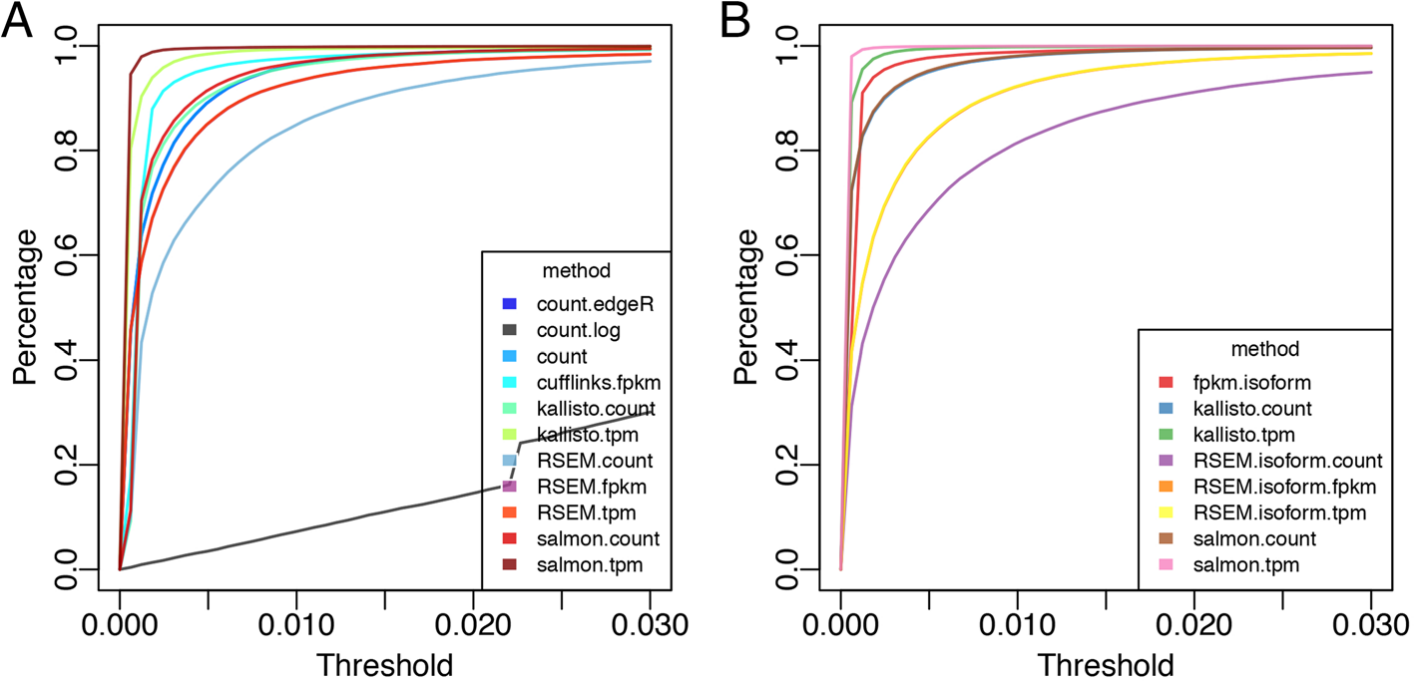
ROC-like curve evaluating linearity of quantified abundance at gene level (**a**) and isoform level (**b**) based on residuals from model *C*∼*m*×*A*+*n*×*B*+*ε*. Proportion of variables with residuals *smaller* than a threshold is computed

In the concordance analysis, we evaluated if the rank of genes from the fitted value *C* is consistent with the rank from the true observed *C*. Plots of the rank of *C* against the rank of $\hat {C}$ in Fig. 4 a demonstrate that count data provide the best concordance while the Cufflink’s FPKM result in the worst concordance. In addition, the log transformation of count data induces a slight underestimation. Furthermore, the concordance of abundance estimated from Kallisto and Salmon enhanced tremendously as the expression level increases. Although good concordance is observed in abundances quantified by some methods at gene level (Fig. 4 a), concordance of abundance at isoform levels is poor in all methods (Fig. 4 b). The poor concordance of isoform level data might due to the bias introduced by isoform abundance quantification methods. Moreover, the concordance analysis (Fig. 2 a) on measured *C* against constructed *C* based on ground truth (0.75×*A*+0.25×*B*)represents consistent result to the concordance analysis between *C* against $\hat {C}$ (Fig. 4 a). In the ROC-like analysis of absolute residual values, we evaluated the proportions of genes or isoforms with residual at a given threshold level t. Results on the abundance at the gene level show that all studied methods, except for log transformation of count, result in reasonable performance. Among which, TPM from Salmon and Kallisto perform the best (Fig. 5 a). Similarly, TPM from Salmon and Kallisto gives the best performance at isoform level and count data perform poorly in general (Fig. 5 b)

**Fig. 6:**
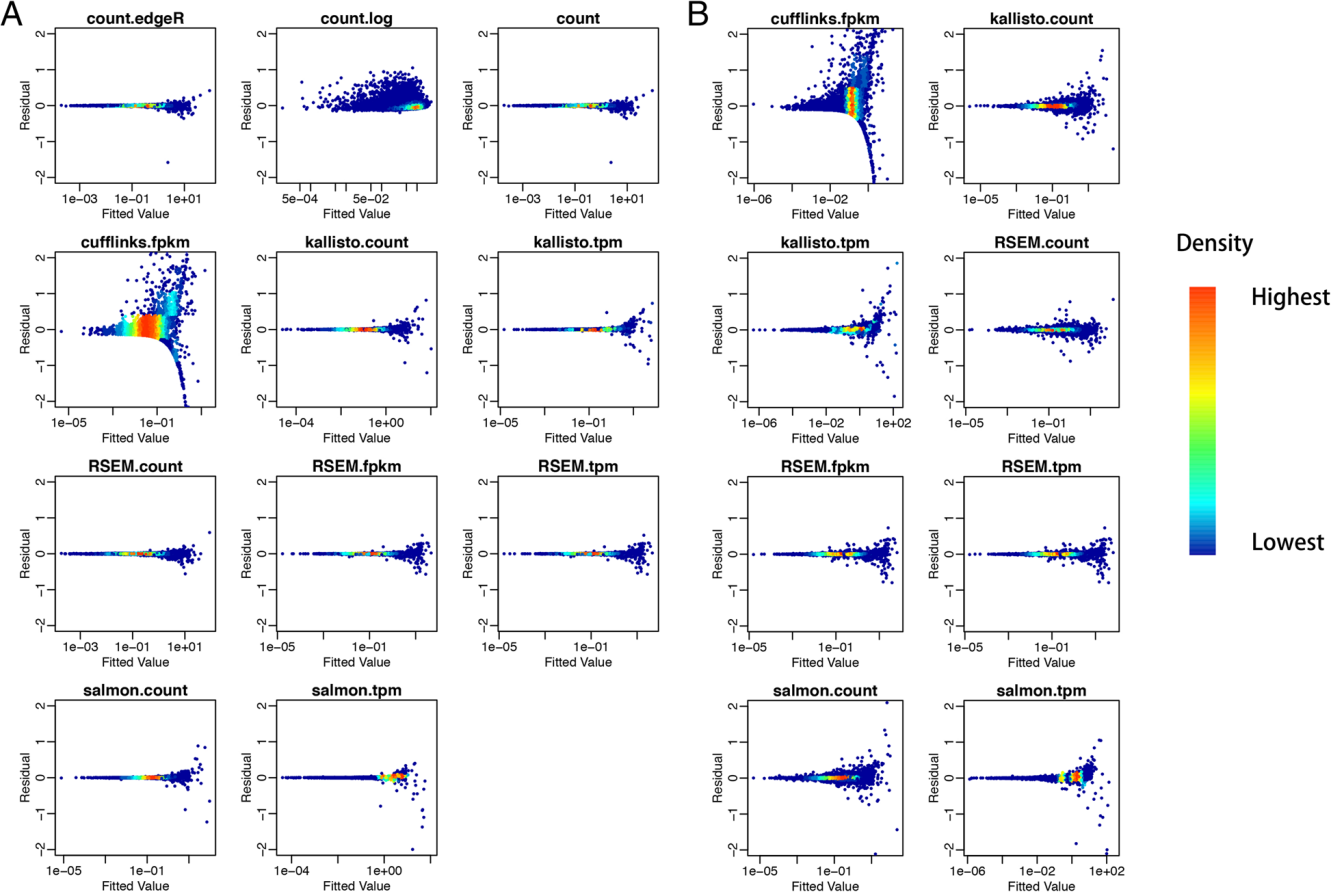
Residual plot for rescaled model $\frac {C-\mu _{C}}{\sigma _{C}}\sim m\times \frac {A-\mu _{A}}{\sigma _{A}} + n\times \frac {B-\mu _{B}}{\sigma _{B}} + \epsilon $ at gene level (**a**) and isoform level (**b**)

In the residual analysis, in order to make the residual comparable across quantification methods in different ranges, we rescaled quantifications and use the model $\frac {C-\mu _{C}}{\sigma _{C}}\sim m\times \frac {A-\mu _{A}}{\sigma _{A}} + n\times \frac {B-\mu _{B}}{\sigma _{B}} + \epsilon $ to conduct multiple linear regression. From the residual plots, we could observe that all the methods except for Cufflinks and log transformation resulted in small residuals across fitted values (Fig. 6 a).

## Discussion

From the results in Fig. 3, we observed that the performance of linear model on count data is largely affected by the library size of samples fitted. Therefore, we suggest normalization as a compulsory preprocess step prior the deconvolution analysis. The poor estimation of isoform based data (Figs. 2, 3, 4, 5 and 6) might due to the challenge of isoform abundance quantification. In addition to the analyses presented in the “Results” section above, we also conducted the same set of analyses on sample *D*, using the model *D*∼*m*×*A*+*n*×*B*+*ε*. Since *D* is made up of A and B with proportion 1:3, the expected value for *m* and *n* is 0.25 and 0.75 respectively in this D-based model. All of the results from the D-based model are consistent with the findings observed from the *C*-based model discussed (Additional file 1: Figure S1, Additional file 2: Figure S2, Additional file 3: Figure S3, Additional file 4: Figure S4 and Additional file 5: Figure S5). Therefore, we can conclude that the evaluation of linearity of quantification data is not affected by the specific proportion of the original mixture. In summary, the main objective of this study is to provide reference to the researcher considering deconvolution as downstream analysis of RNA-Seq data. Although linear assumption is the priority of deconvolution analysis, it does not guarantee the performance. For future study, assessment of deconvolution performance is still required.

## Conclusions

We conducted a comprehensive study to assess the linearity of gene and isoform abundance reported by different RNA-seq quantification methods based on the performance how these quantifications fitted in a multiple regression linear model. From our analysis, we observed that abundance at both the gene and isoform level from different quantification methods exhibit distinct distribution patterns and thus give diverse results. Abundance reported in the units of counts gave poor linearity, demonstrated from the worst estimated parameters and intercept values of the model. This indicates the necessity of normalizing the abundance data prior the deconvolution analysis. In total, TPM reported by Salmon and Kallisto is the best abundance data for linear models as the estimated parameters are close to true mixture proportions and the fitted values are tightly linearly correlated to the sample’s measured abundance. Moreover, when comparing within the same quantification method at both gene and isoform levels, the correlation between the measured and models’ fitted values is lower at isoform level compared to gene level while the performance on estimating parameters of linear models are similar in both levels.

## Methods

### Dataset

The dataset used in this study is the RNA-seq data generated by the Sequencing Quality Control (SEQC) project. Raw data in fastq format were obtained from GEO website with accession number GSE47774. List of downloaded samples and their details is specified in Additional file 6: Table S1.

### Data quantification and analysis

Raw sequence reads from multiple lanes were first merged into one for each replicate of each sample. To prepare sequence map files for alignment-based quantification methods, the merged sequenced reads were mapped to human reference genome (hg19) using Tophat2 (version 2.1.0) with default parameters. The mapped files were then used by quantification methods HTSeq (version 0.6.1) to obtain count data, Cufflinks (versin 2.1.1) to obtain FPKM data. RSEM (version 1.2.31) will first mapped the raw reads using its default aligner Botwtie2 (version 2.1.0) onto human genome (hg19) and then estimate count, FPKM, and TPM. EdgeR (version 3.12.1) is used for count data transformation. Alignment-free quantification methods Kallisto (version 0.42.5) and Salmon (version 0.6.0) estimate the count and TPM data based on index built from human transcriptome (GRCh37). All the quantification methods were run at both the gene and isoform levels. All the statistical analysis and plots were carried out in R environment (version 3.2.3).

## Additional files

Additional file 1
**Figure S1.** Concordant analysis between rank of quantifications of $0.25 	\times \bar{A} + 0.75 	\times \bar{B}$(Constructed Value) and $\bar{D}$ (Measured Value) at gene level (a) and isoform level (b). Rankes were normalized by the number of quantifications in each plot. (PDF 6230 kb)

Additional file 2 **Figure S2.** Jitter boxplot of estimated coefficients and intercepts from linear model *D*∼*m*×*A*+*n*×*B*+*ε* at gene level (a) and isoform level (b). Red line indicates expected estimates if *D*, *A* and *B* satisfy linear assumption. (PDF 1520 kb)

Additional file 3 **Figure S3.** Concordant analysis between rank of estimated quantifications and rank of measured abundance value at gene level (a) and isoform level (b). The fitted value in the y-axis is estimated from model *D*∼*m*×*A*+*n*×*B*+*ε*. Ranks were normalized by the number of quantifications in each plot. (PDF 5950 kb)

Additional file 4 **Figure S4.** ROC-like curve evaluating linearity of quantified abundance at gene level (a) and isoform level (b) based on residuals from model *D*∼*m*×*A*+*n*×*B*+*ε*. Proportion of variables with residuals smaller than a threshold is computed. (PDF 1160 kb)

Additional file 5
**Figure S5.** Residual plot for rescaled model $\frac{D-\mu_{D}}{\sigma _{D}}\sim m\times\frac{A-\mu _{A}}{\sigma _{A}} + n\times\frac{B-\mu _{B}}{\sigma _{B}} + \epsilon $ at gene level (a) and isoform level (b). (PDF 2990 kb)

Additional file 6 **Table S1.** Column 1 and 2 show the sample type and replicates index; Column 3 shows total read pairs, column 4-7 show the mapping rate from different quantification methods (Tophat, RSEM, Kallisto and Salmon). (XLS 61 kb)

## Abbreviations

EM: Expectation maximization
FPKM: Fragments per kilobase of transcript per million mapped reads
NVS: Norvartis
ROC: Receiver operating characteristics
RPKM: Reads per kilobase of transcript per million mapped reads
SEQC: Sequencing qualify control
TPM: Transcripts per million
