# Supplementary figures and images for: Comprehensive evaluation of RNA-seq quantification methods for linearity
Source: BMC Bioinformatics. 2017 Mar 22;18(Suppl 4):117. doi: 10.1186/s12859-017-1526-y (PMC5374695; doi:10.1186/s12859-017-1526-y)

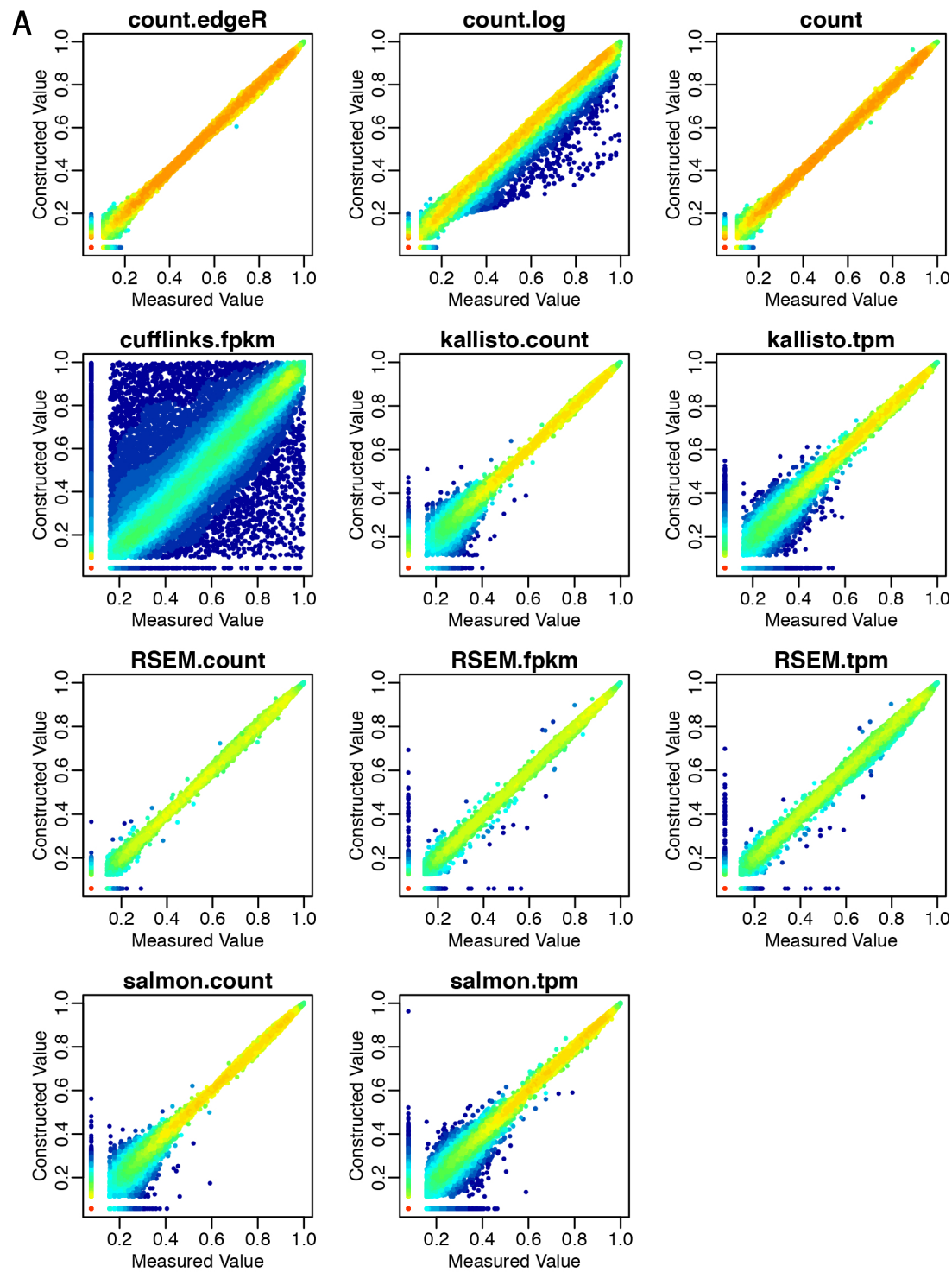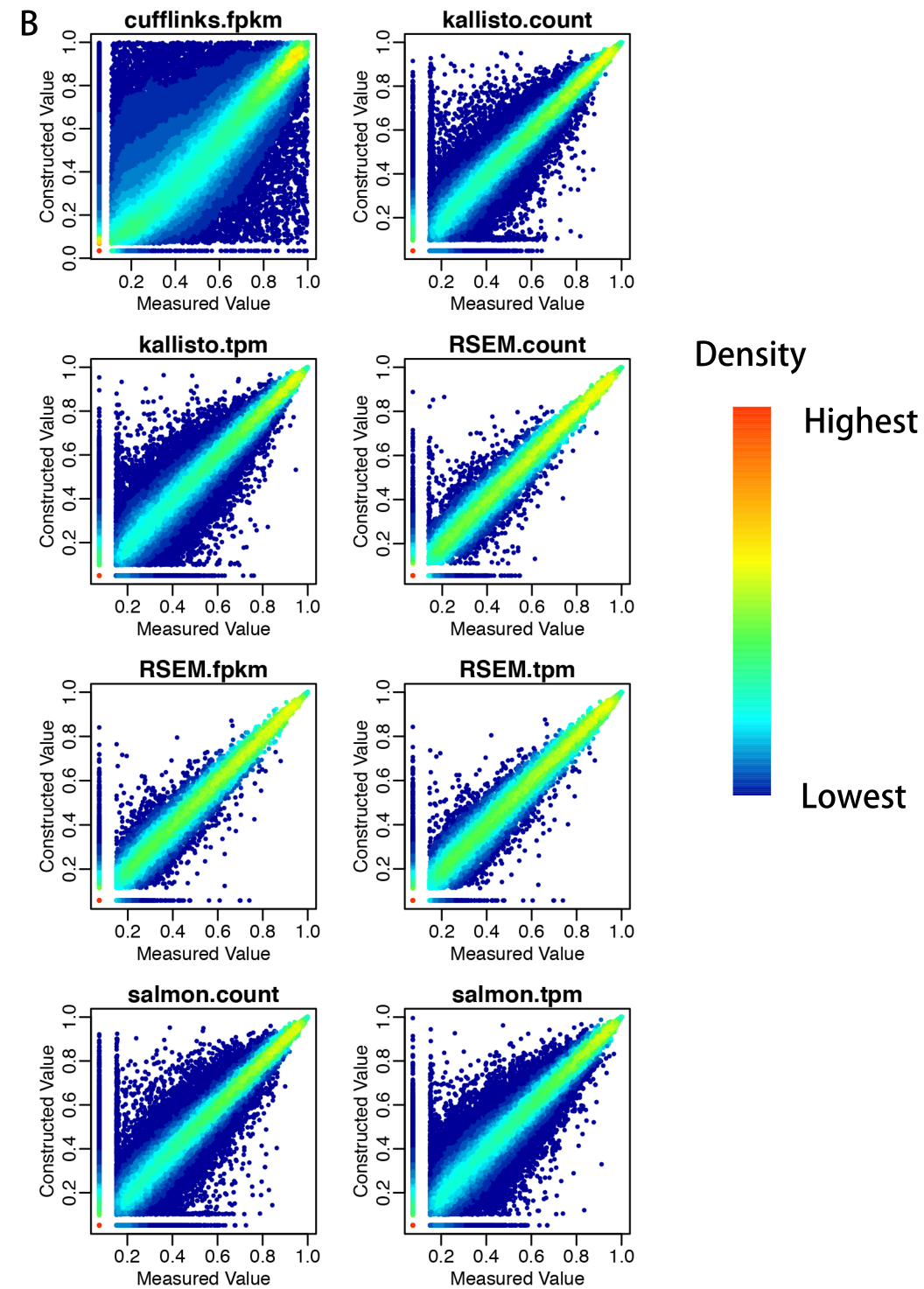

Supplement: Supplementary file 1 — Figure S1. Concordant analysis between rank of quantifications of $0.25 \times \bar{A} + 0.75 \times \bar{B}$0.25×Ā+0.75×B¯(Constructed Value) and $\bar{D}$D¯ (Measured Value) at gene level (a) and isoform level (b). Rankes were normalized by the number of quantifications in each plot. (PDF 6230 kb) [file 12859_2017_1526_MOESM1_ESM.pdf]

**A**

Intercept

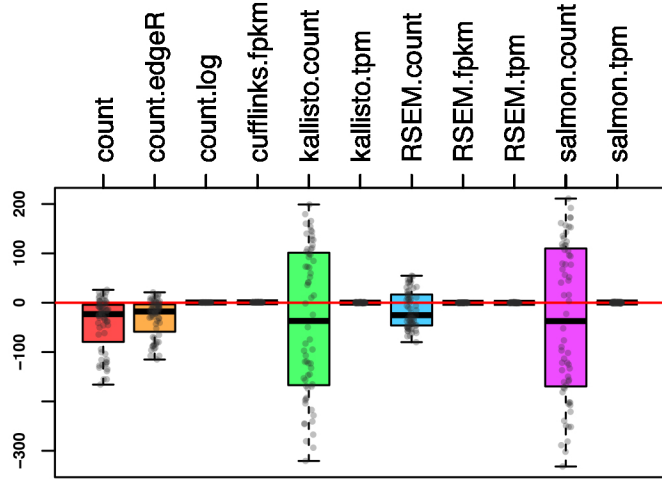

Parameter1

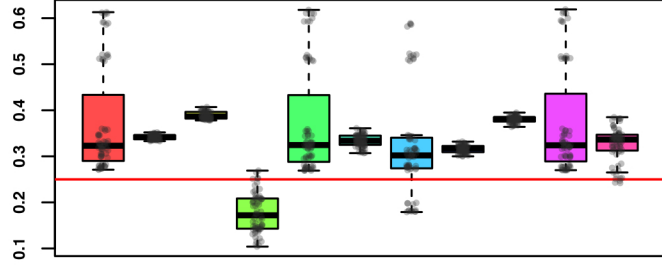

Parameter2

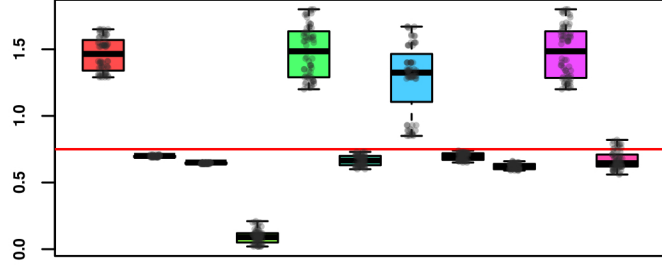**B**

Intercept

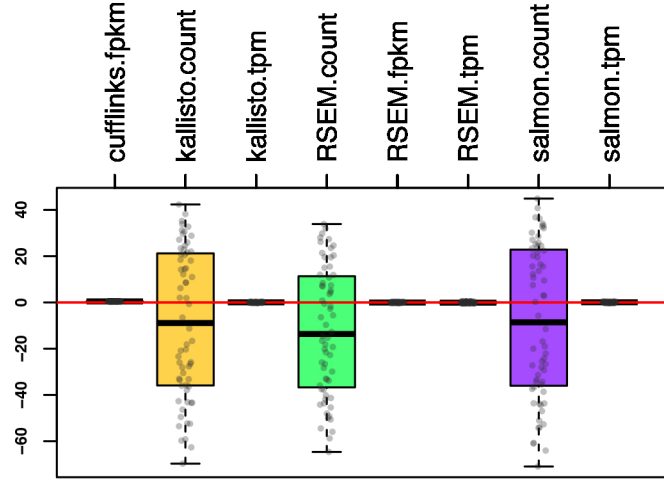

Parameter1

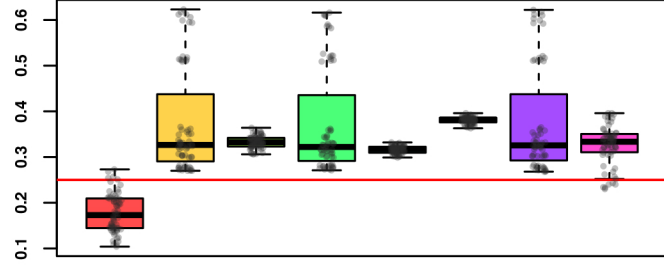

Parameter2

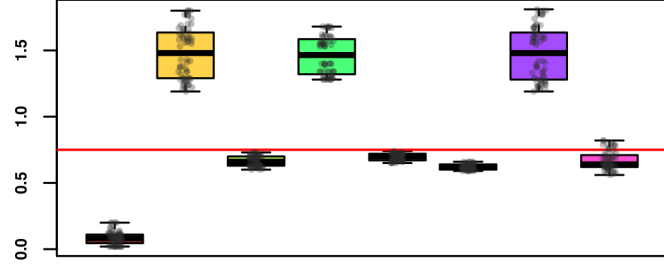

Supplement: Supplementary file 2 — Figure S2. Jitter boxplot of estimated coefficients and intercepts from linear model D∼m×A+n×B+ε at gene level (a) and isoform level (b). Red line indicates expected estimates if D, A and B satisfy linear assumption. (PDF 1520 kb) [file 12859_2017_1526_MOESM2_ESM.pdf]

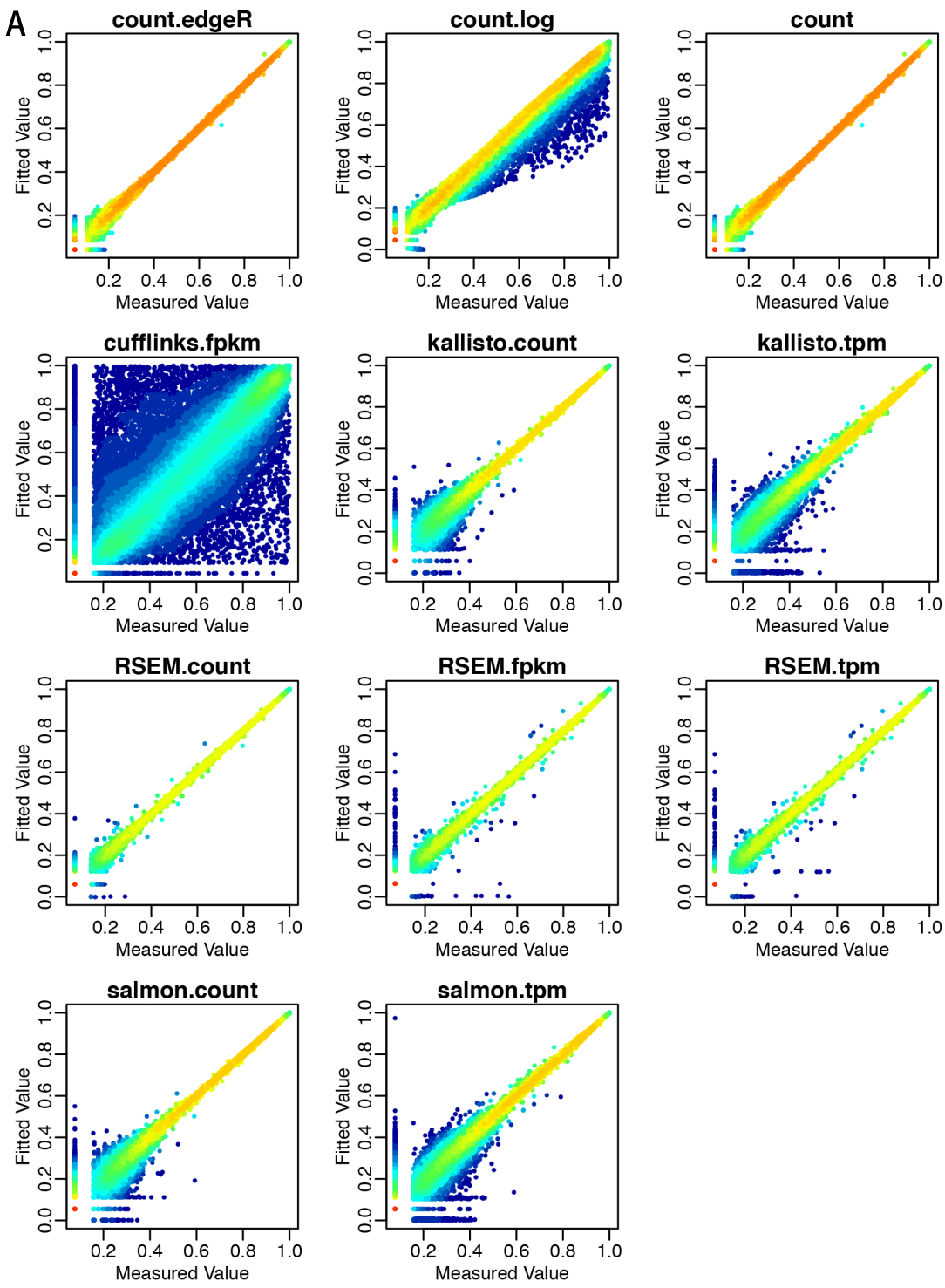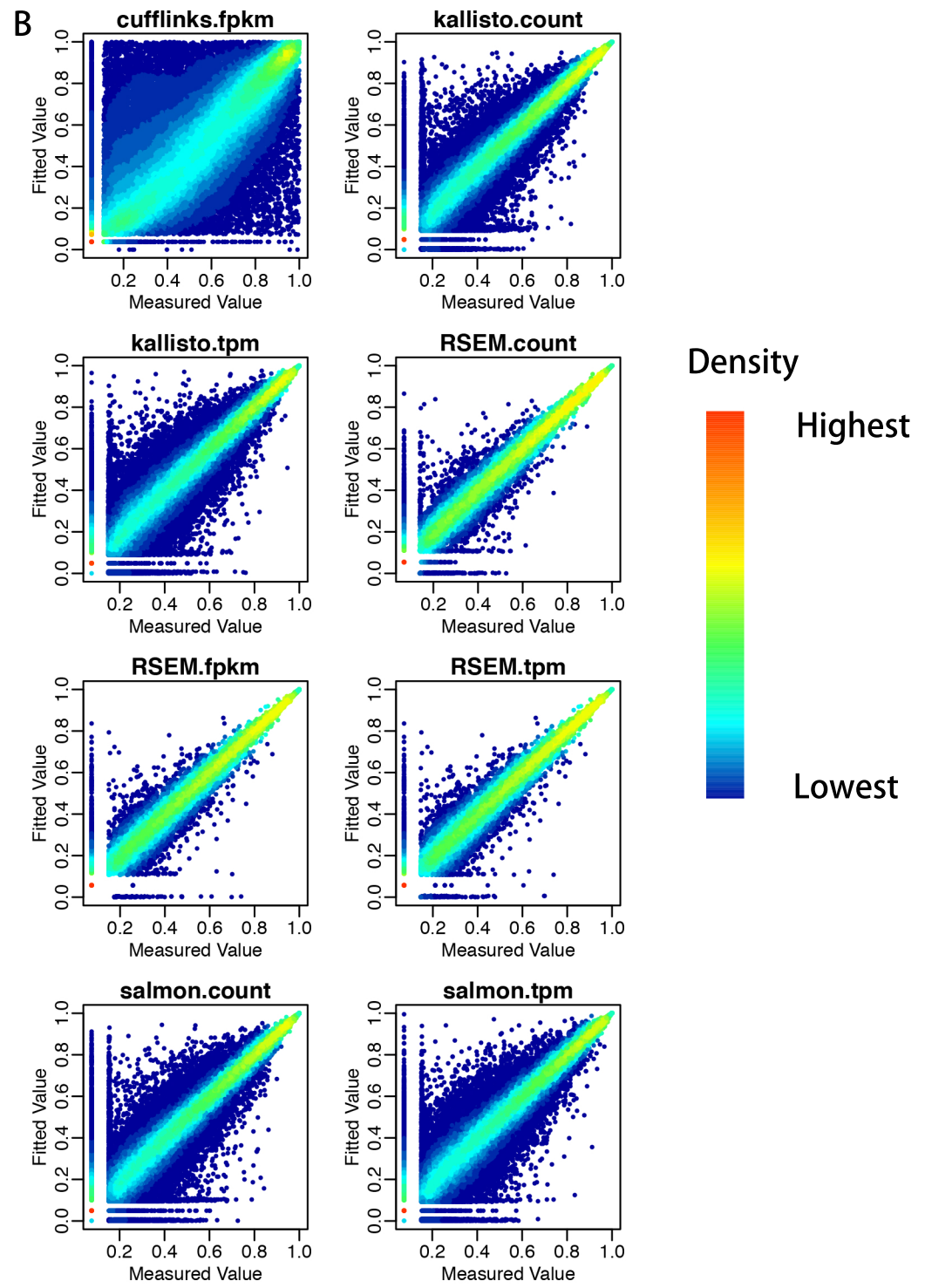

Supplement: Supplementary file 3 — Figure S3. Concordant analysis between rank of estimated quantifications and rank of measured abundance value at gene level (a) and isoform level (b). The fitted value in the y-axis is estimated from model D∼m×A+n×B+ε. Ranks were normalized by the number of quantifications in each plot. (PDF 5950 kb) [file 12859_2017_1526_MOESM3_ESM.pdf]

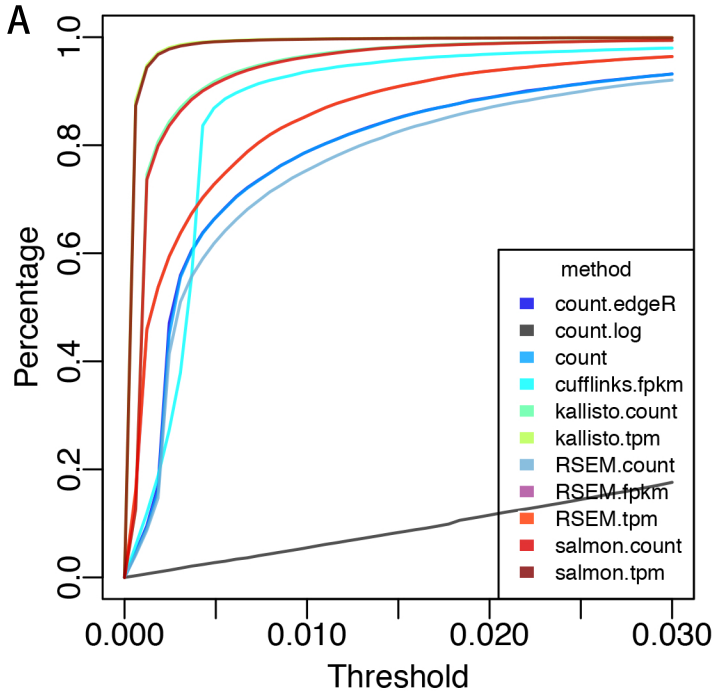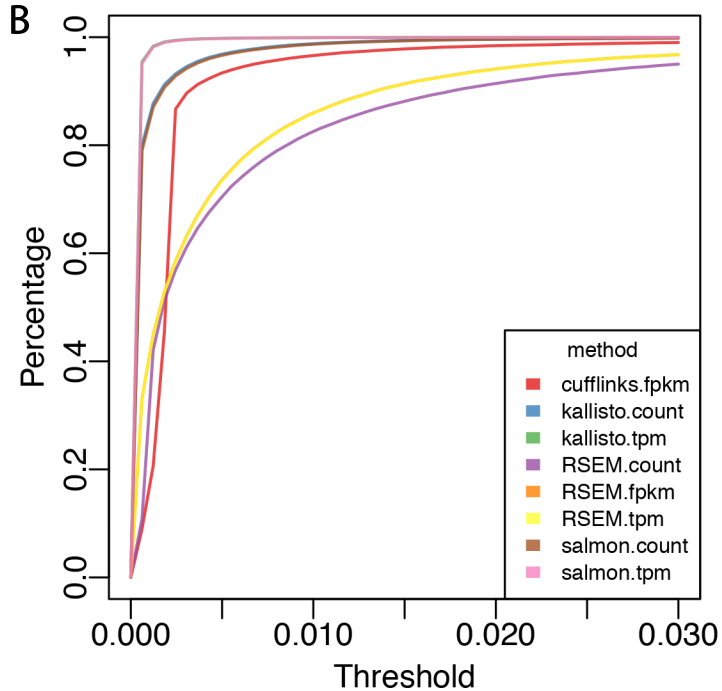

Supplement: Supplementary file 4 — Figure S4. ROC-like curve evaluating linearity of quantified abundance at gene level (a) and isoform level (b) based on residuals from model D∼m×A+n×B+ε. Proportion of variables with residuals smaller than a threshold is computed. (PDF 1160 kb) [file 12859_2017_1526_MOESM4_ESM.pdf]

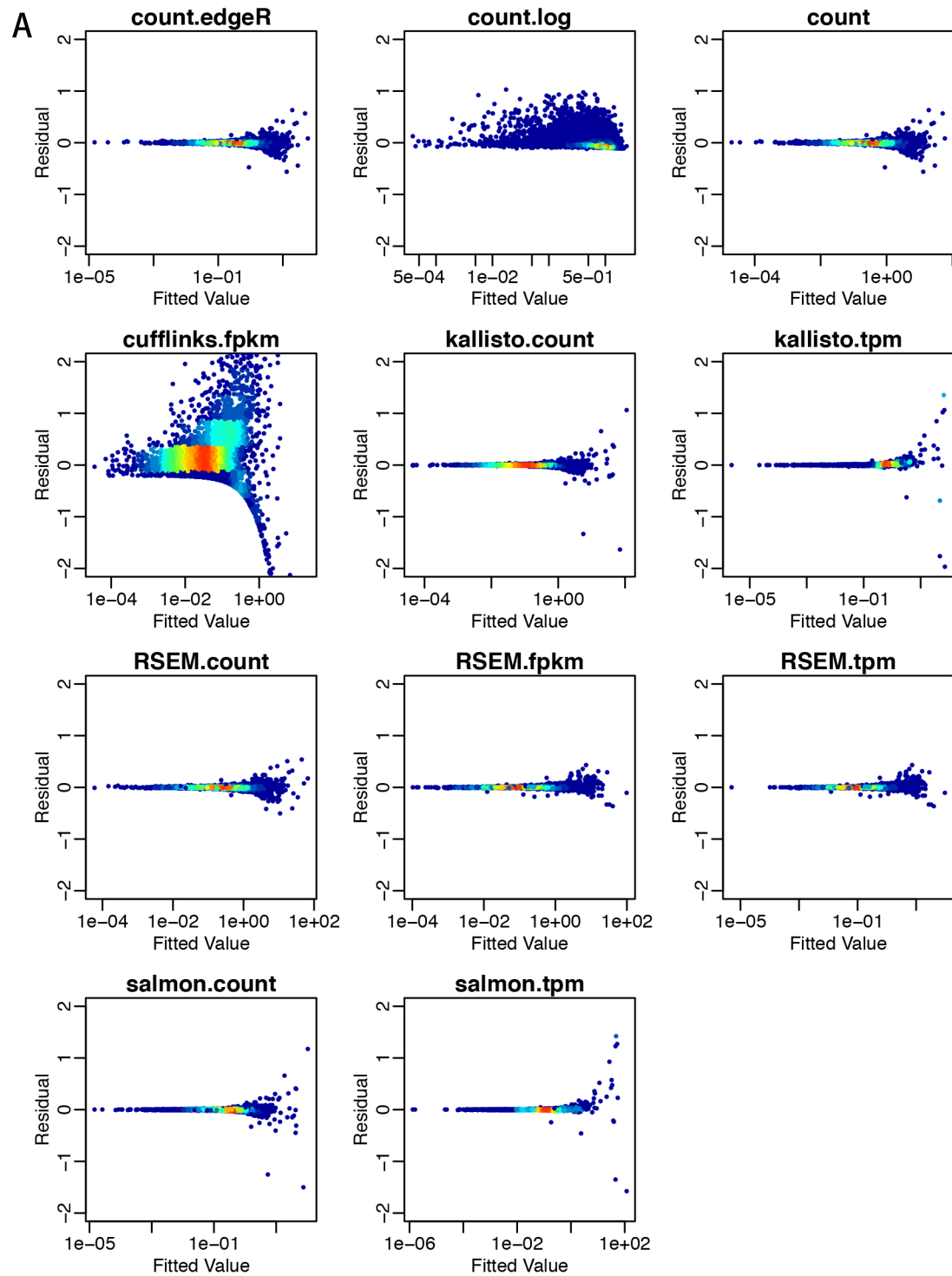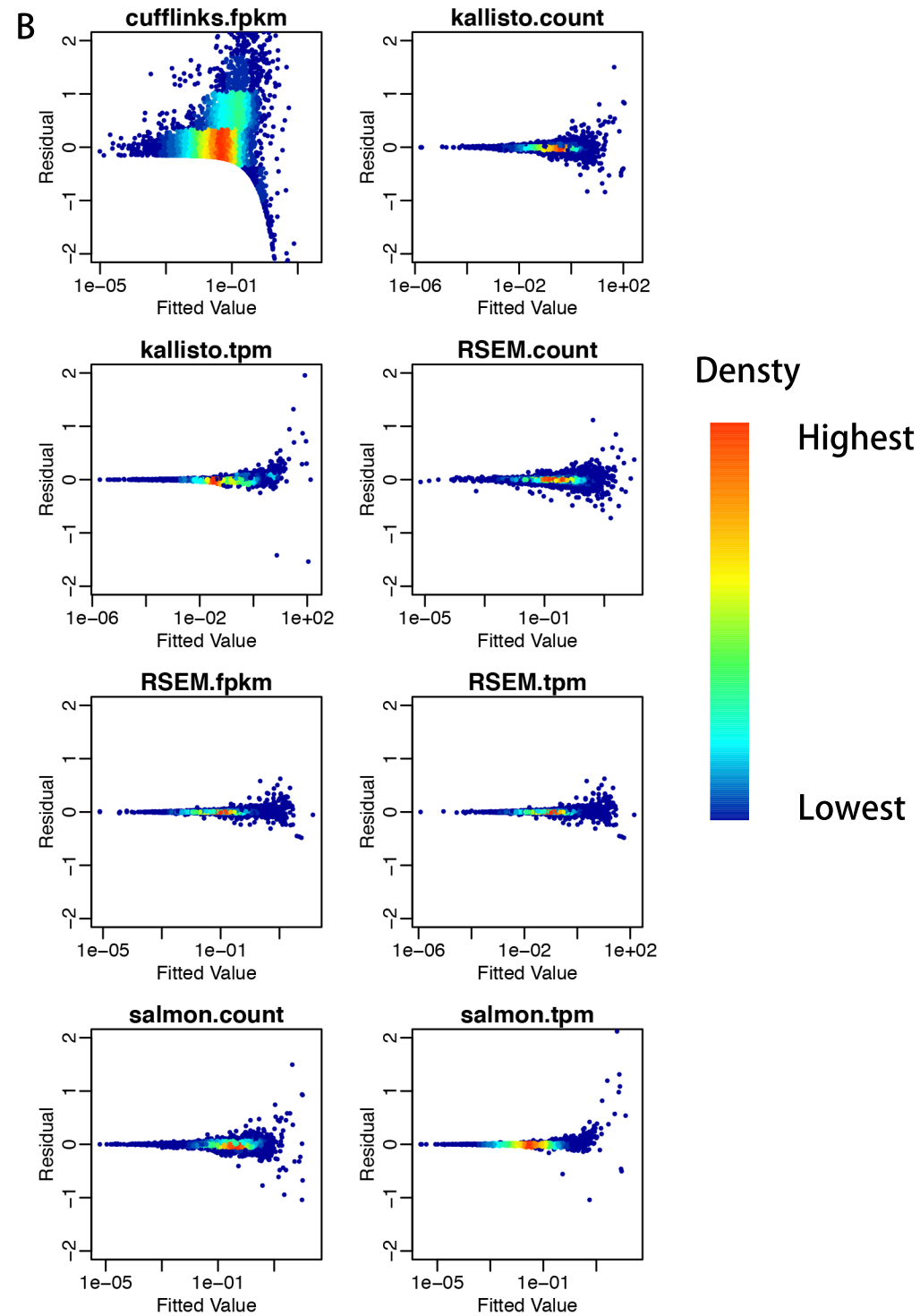

Supplement: Supplementary file 5 — Figure S5. Residual plot for rescaled model $\frac{D-\mu_{D}}{\sigma _{D}}\sim m\times\frac{A-\mu _{A}}{\sigma _{A}} + n\times\frac{B-\mu _{B}}{\sigma _{B}} + \epsilon$D−μDσD∼m×A−μAσA+n×B−μBσB+ε at gene level (a) and isoform level (b). (PDF 2990 kb) [file 12859_2017_1526_MOESM5_ESM.pdf]
